# Analysis of the Longitudinal Association Between Parental Feeding Practices and Body Composition Among Children in Shenzhen

**DOI:** 10.3390/nu17142255

**Published:** 2025-07-08

**Authors:** Sha Liu, Chao Li, Dingkang Wang, Bizhong Che, Weimin Liu, Wei Xia, Wei Wei, Youfa Wang

**Affiliations:** 1 Global Health Institute, School of Public Health, Xi’an Jiaotong University Health Science Center, Xi’an Jiaotong University, Xi’an 710049, China; liusha09152022@163.com (S.L.); lc18182582951@163.com (C.L.); dkwang@xjtu.edu.cn (D.W.); boche@xjtu.edu.cn (B.C.); 2 Nuclear Industry 215 Hospital of Shaanxi Province, Xianyang 712000, China; 3 Luohu District Center for Disease Control and Prevention, Shenzhen 518001, China; youjie-45@126.com (W.L.); lhcdcspk@163.com (W.X.); 4 Institute of Health Sciences, China Medical University, Shenyang 110122, China; wei378023020@gmail.com

**Keywords:** parental feeding practices, obesity, body composition, children

## Abstract

**Background**: A national study from China in 2023 predicted that the prevalence of overweight and obesity among children aged 7–18 will increase from 23.4% in 2019 to 32.7% by 2030. **Objectives**: This study aimed to investigate the longitudinal association between parental feeding practices and children’s body composition and weight status, and to assess the mediation effect of parental feeding practices in the relationship between socioeconomic status and childhood body composition. **Methods**: This longitudinal observational study was conducted between September and November 2021 in eight primary schools located in Luohu District, Shenzhen. Baseline and two follow-up surveys were administered annually during the same period from 2021 to 2023 (with one-year intervals). A total of 620 third-grade students (aged 8–10 years at baseline) and their parents were ultimately included in the study. Associations between parental feeding practices and children’s weight status and body composition were analyzed using mixed-effects models. The mediation effect of parental feeding practices on the relationship between socioeconomic status and childhood body composition was assessed through bootstrapping analysis. **Results**: At follow-up, a significant upward trend in the prevalence of central obesity among children was observed. Among all parental feeding dimensions, perceived child weight (PCW) demonstrated a strong association with central obesity after Bonferroni correction (OR = 1.33, 95% CI = 1.16, 1.51); similarly, monitoring (MN) and concern about child weight (CN) were both significantly associated with central obesity as risk factors for central obesity (OR = 1.14, 95% CI = 1.06, 1.23; OR = 1.16, 95% CI = 1.07, 1.27), both *p* < 0.001. These associations were modified by baseline child sex, parental BMI, and maternal and paternal education levels. However, restriction (RST) was not significantly associated with either body composition or weight status. The relationship between family socioeconomic status (SES) and childhood overweight and obesity was mediated by pressure to eat (PE) (*p* < 0.05). **Conclusions**: MN, PCW, and CN are associated with an increased risk of obesity in children. However, no significant association was found between parental feeding practices and changes in children’s body composition.

## 1. Introduction

The growing burden of childhood nutrition and health has become a major global public health concern, with childhood overweight and obesity representing one of the most pressing challenges in this field [1,2]. According to a 2016 report by the World Health Organization (WHO), over 340 million children and adolescents aged 5–19 years were overweight or obese, with the standardized prevalence of obesity among girls increasing from 0.7% to 5.6% and among boys from 0.9% to 7.8% [3]. The World Obesity Federation predicts that by 2035, 383 million children and adolescents aged 5–19 will be overweight or obese [4]. Since 2000, the average body mass index (BMI) in many high-income countries has plateaued, frequently remaining at elevated levels, while BMI in low- and middle-income countries continues to rise. The China Child Obesity Report highlights a continuous rise in the prevalence of childhood overweight and obesity. A 2023 study in China predicted that the prevalence of overweight and obesity among children and adolescents aged 7–18 will increase from 23.4% in 2019 to 32.7% by 2030 [5]. The hazards of childhood obesity primarily encompass persistent obesity into adulthood, increased risks of type 2 diabetes, cardiovascular disease, chronic kidney disease, cancer, and elevated mortality rates [6].

BMI remains currently the most widely utilized clinical standard for evaluating health status and screening for overweight and obesity [7,8]. However, BMI does not directly estimate the distribution of various body composition components, such as body fat, muscle mass, and body water [9], which may result in inaccuracies in the assessment of childhood overweight and obesity [10]. Therefore, the use of BMI for obesity screening has inherent limitations. Recent studies have shown that the waist-to-height ratio (WHtR) is a more accurate predictor of central obesity and associated metabolic risks compared to BMI, with a WHtR ≥ 0.48 indicating an elevated risk of central obesity in children. WHtR has been demonstrated to be a stronger predictor of elevated morbidity, such as type 2 diabetes and hypertension, than BMI-based classifications [11]. Furthermore, body composition analysis (BCA) has been recognized as an objective and reliable method for assessing body fat content and distribution, offering valuable guidance for obesity screening as well as for the prevention of obesity-related complications and metabolic diseases [12].

Numerous studies have shown that fat mass index (FMI) and fat-free mass index (FFMI) are closely associated with childhood obesity, and serve as valuable predictors of children’s health outcomes [13,14,15]. Parental beliefs, attitudes, and feeding strategies may affect childhood obesity by influencing children’s eating behaviors and nutritional intake. Specifically, parental approaches such as food monitoring (MN), restriction (RST), and pressure to eat (PE) have been shown to impact children’s dietary habits and weight-related outcomes [16]. In low-income households, restrictive feeding behaviors by parents are associated with an increased risk of obesity in children later in life. This restriction feeding is often accompanied by the availability of energy-dense, nutrient-poor foods, which may paradoxically lead to weight gain rather than weight loss in children [17]. Inappropriate parental feeding practices can disrupt normal feeding processes and hinder the establishment of healthy eating behavior patterns in children.

Children’s growth and development are strongly influenced by their family socioeconomic status (SES), which is a key determinant of health and nutritional outcomes. SES is typically evaluated based on educational level, income level, and occupational status [18]. In developed countries, SES is inversely correlated with the prevalence of obesity, while in developing countries, SES shows a positive correlation with the prevalence of overweight and obesity [19]. Research indicates that SES may affect children’s weight status by influencing parental feeding practices. SES-related differences in parental education, occupation, and household income may contribute to variations in feeding practices, which in turn shape children’s dietary patterns and access to nutrient-rich foods. This coping mechanism may result in excessive energy intake, which, over the long term, increases the risk of overweight and obesity [20].

Although previous cross-sectional studies have reported associations between parental feeding practices and children’s BMI, there is still a lack of research on their longitudinal associations. Our longitudinal design addresses this gap by adjusting for baseline BMI, thereby enabling a more robust assessment of temporal effects. The objectives of this study are to further explore the association between parental feeding practices and childhood body composition in Shenzhen, including nutritional status indicators and body composition. Through longitudinal repeated measurements, we hypothesized that parental feeding practices may predict changes in children’s body composition after adjusting for baseline BMI, and that the mediation effect of parental feeding practices may influence the relationship between socioeconomic status and childhood body composition.

## 2. Materials and Methods

### 2.1. Study Design and Participants

This longitudinal observational study was conducted with support from the Children’s Nutrition Project in Luohu District, Shenzhen. A cluster-randomized sampling approach was employed to recruit participants. At baseline, a total of 957 third-grade students from eight primary schools in the Luohu District were enrolled between September and December 2021. Baseline data were collected on children’s body composition, anthropometric measures, sociodemographic characteristics, parental feeding behaviors, and family SES indicators. Annual follow-up surveys were conducted during the same period in 2022 and 2023, collecting the same data as the baseline. After excluding subjects with missing variables (e.g., body composition), 620 students (ages 8–10 years) were included. The project was reviewed and approved by the Ethics Committee of the Luohu District Center for Disease Control and Prevention in Shenzhen (Approval Number: Luojilunzi [2021] No. 001A) and was conducted after obtaining written informed consent from the students and their parents.

### 2.2. Questionnaire Survey and Physical Measurements

Questionnaire survey: the parent questionnaire was administered online and completed by children’s mothers or other primary caregivers, which collected information on their sociodemographic characteristics, family characteristics, parental feeding practices and attitudes, and anthropometrics at home. Parental feeding practices, which are part of the parent questionnaire, were assessed using a modified 26-item scale derived from the Child Feeding Questionnaire (CFQ). The children’s questionnaire was completed by children under the guidance of professionals in the classroom, which collected information on children’s sociodemographic characteristics and eating behaviors.

Anthropometric measurements: Students’ height and weight were measured using the HGM-700 height and weight scale, with participants barefoot and wearing light clothing. Measurements were recorded to the nearest 0.1 kg after the values stabilized. BMI was calculated as weight (kg) divided by height squared (m^2^). Waist circumference was measured using a non-elastic soft tape measure, with the student standing naturally, abdomen relaxed, and breathing evenly. The tape was positioned 1 cm above the umbilicus, passing horizontally around the body at the midpoint between the lower margin of the 12th rib and the iliac crest. Readings were taken at the end of a calm exhalation, accurate to 0.1 cm. Body composition was measured using the TANITA MC-780MA (TANITA Corporation, Shanghai, China) body composition analyzer, strictly following the manufacturer’s operating instructions [21]. Parental height and weight were self-reported via questionnaire.

### 2.3. Outcome Variables

Main outcome: Body composition indices were measured using eight-electrode bioelectrical impedance analysis (BIA) technology (TANITA MC-780MA), providing indices such as body fat mass (BFM) and fat mass percent (BFP) [22]. Principal component analysis (PCA) was employed to identify fat mass index (FMI) and fat-free mass index (FFMI) as the two primary indicators of body composition in this study. FMI was calculated as BFM divided by height squared (kg/m^2^), and FFMI was calculated as FFM divided by height squared (kg/m^2^). BIA-derived measures (e.g., FMI) rely partially on weight-based equations, potentially limiting precision.

Secondary outcome: Overweight and obesity were defined based on the participant’s BMI, using the Chinese national standard “WS/T 586-2018: Screening for Overweight and Obesity among School-aged Children and Adolescents” [23]. Overweight and obesity are defined based on age- and sex-specific BMI percentiles: underweight/normal weight (<85th percentile), overweight (≥85th percentile and <95th percentile), and obesity (≥95th percentile). Central obesity was assessed using the waist-to-height ratio (WHtR), calculated as waist circumference (m) divided by height (m), with a WHtR ≥ 0.48 defining central obesity [24].

### 2.4. Exposure Variables

Parental feeding practices were assessed using a modified 26-item scale derived from the Child Feeding Questionnaire (CFQ) [25]. The CFQ comprises seven dimensions, namely: Monitoring (MN), Pressure to Eat (PE), Restriction (RST), Perceived Child Weight (PCW), Concern About Child Weight (CN), Perceived Parent Weight (PPW), and Food as Reward (FR). The Child Feeding Questionnaire scale has demonstrated good internal consistency, with Cronbach’s α coefficient being 0.70~0.92, which has demonstrated good validity and reliability among Chinese children. The CFQ primarily evaluates parental feeding practices characterized as negative or controlling, but does not encompass assessments of positive feeding behaviors. The primary caregiver completed the scale using a five-point Likert response format, with each point anchored by descriptive verbal labels.

### 2.5. Covariates

Child and parental characteristics were included as covariates in the analysis. These variables encompassed age (years), gender (male or female), survey year, parent BMI, and parent education level (middle school or below, high school or vocational school, or university or above), as well as monthly household income. Gender and parental education levels were also utilized as potential influencing factors for stratified analysis [26].

### 2.6. Statistical Methods

Normality of continuous variables was assessed using the Shapiro-Wilk test. Chi-square tests (for categorical variables) and ANOVA (for continuous variables) were utilized to examine the baseline differences in children’s and parents’ characteristics according to parental feeding practices [27]. Mixed-effects models were adopted to statistically test the associations between parental feeding practices and childhood body composition indicators [28]. To account for multiple comparisons, we applied the Bonferroni correction by adjusting the significance level to 0.05 divided by the number of comparisons (e.g., 0.05/28 ≈ 0.001 for the seven dimensions of parental feeding practices).

**Figure 1:**
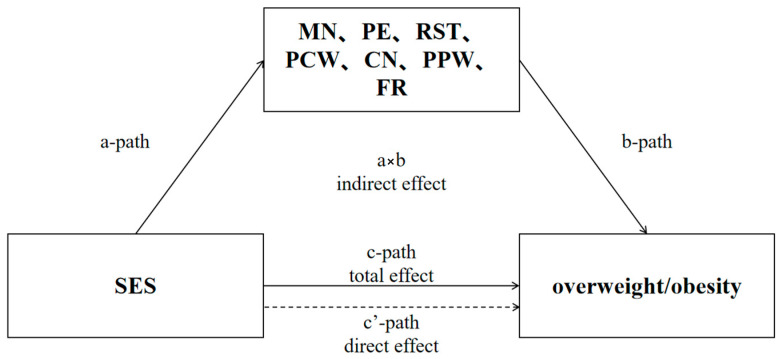
Intermediate path.

A mediation model was employed to examine the individual mediating effects. The outcome variable was constructed as a childhood overweight and obesity indicator at the second follow-up. The mediator variable was parental feeding practice pattern at the first follow-up. The exposure variable was the household socioeconomic indicator at baseline. The control variables were child sex and maternal and paternal education, and BMI. Bootstrapping was employed to assess the statistical significance of the mediating effect of parental feeding practices, based on the 95% bias-corrected confidence interval (CI) obtained from the bootstrapping (Figure 1). Data management and statistical analyses were performed using STATA version 17.0 (StataCorp, College Station, TX, USA). A two-tailed *p*-value < 0.05 indicated a statistically significant difference.

### 2.7. Quality Control

All investigators underwent uniform training [29]. The questionnaire was developed based on a comprehensive literature review and consultations with senior experts in the field, aligned with the study objectives. The Cronbach’s α coefficient of the Child Feeding Questionnaire (CFQ) was 0.72, indicating good reliability and validity. Investigators were trained to clarify the research objectives, and specific implementation plans, familiarize themselves with the survey process, and formulate solutions for potential issues. All survey tools were uniformly provided by the survey team. Double verification was implemented for both the questionnaire and laboratory test results. Inconsistent parts were reviewed, confirmed, and modified accordingly.

## 3. Results

### 3.1. Demographic Characteristics and Health Outcomes in Parental Feeding Practices

The mean age of male children was 8.79 ± 0.02 years, while that of female children was 8.76 ± 0.02 years. A significant upward trend in the prevalence of both overweight/obesity and central obesity was observed from the lower to the higher quartiles of concern scores (both *p* < 0.001). Fathers with an educational level of university or above exhibited significantly higher scores in PE and CN. Notably, children whose caregivers exhibited higher PE scores demonstrated a significantly lower prevalence of overweight/obesity compared to those with lower PE scores. Conversely, children with higher scores in PCW, CN, and PPW showed significantly higher prevalence of overweight/obesity compared to those with lower scores (Table 1). Significant differences in the prevalence of overweight/obesity, obesity, and central obesity were noted across varying scores of PE, PCW, CN, and PPW (all *p* < 0.001). However, there were differences in CN scores concerning parental education and household monthly income (all *p* < 0.05), while no significant differences were observed in other dimensions of parental feeding practices (Table S1).

### 3.2. Distribution of Childhood Overweight and Obesity in Family Socioeconomic Status and Mediating Factors

A total of 620 children were included in the baseline study, among whom 164 were overweight and 456 were non-overweight. The BMI of mothers and fathers of overweight or obese children was higher than that of parents of non-overweight children. Additionally, only the maternal occupation level showed a significant difference between the two groups (*p* < 0.05). However, no significant differences were found in parental education levels or household income (Table S2). Among the mediating factors of parental feeding practices, the scores of PCW (13.23 ± 1.83 vs. 11.01 ± 1.91), CN (7.21 ± 3.00 vs. 4.78 ± 2.40), and PPW (8.79 ± 1.90 vs. 8.17 ± 1.78) for children with overweight or obesity were significantly higher than those for children with non-overweight or obesity (all *p* <0.001). Conversely, the PE score for children with overweight or obesity (11.55 ± 3.50 vs. 13.23 ± 3.45, *p* < 0.001) was significantly lower than that for children with non-overweight (Table S2).

### 3.3. Descriptive Analysis of Baseline and Follow-Up Outcome Indicators

At baseline, among the body composition indicators, FFMI showed significant upward trends over time, with significant gender differences: boys were higher than girls (*p* < 0.001). The prevalence of overweight/obesity and central obesity among children were 26.45% and 22.90%, respectively. From 2021 to 2023, a significant upward trend was observed in the prevalence of overweight/obesity and central obesity (*p* < 0.001) (Table 2).

### 3.4. Association Between Parental Feeding Practices and Children’s Weight Status and Body Composition in Shenzhen

Among the main outcome indicators, an increase in FMI (*β* = 0.04, 95% CI = 0.003, 0.07) was positively associated with MN (both *p* < 0.05). Conversely, a decrease in FMI (*β* = −0.05, 95% CI = −0.10, −0.002, *p* < 0.05) was negatively correlated with FR. An increase in FFMI (*β* = 0.03, 95% CI = 0.01, 0.06) was positively associated with PPW (both *p* < 0.05). Among the body composition indicators, parental feeding practices were positively correlated with weight status indicators. Similarly, the increase in overweight/obesity was positively correlated with MN (OR = 0.12, 95% CI = 0.03, 0.21; *p* = 0.009), PCW (OR = 0.22, 95% CI = 0.05, 0.38; *p* = 0.006), and CN (OR = 0.17, 95% CI = 0.06, 0.28; *p* = 0.003). Similarly, the increase in central obesity was significantly positively associated with PCW (OR = 0.26, 95% CI = 0.12, 0.39) and CN (OR = 0.16, 95% CI = 0.08, 0.25) (both *p* < 0.001). (Table 3).

After applying the Bonferroni correction, the increase in overweight/obesity was not associated with MN, PCW, and CN (all *p* > 0.001). Additionally, PCW (OR = 0.26, *p* < 0.001) and CN (OR = 0.16, *p* < 0.001) were protective against central obesity among all children. However, no significant associations were observed between parental feeding practices and either FMI or FFMI among all children (Table 3).

### 3.5. Mediating Effect of SES on Childhood Overweight and Obesity

Socioeconomic factors of the family were treated as independent variables, while children’s overweight and obesity rates served as dependent variables. A mediation model was constructed, adjusting for potential confounding variables including the child’s age, sex, parental BMI, and parental occupation. Bootstrap analysis revealed that the relationship between socioeconomic factors of the family and children’s overweight was mediated by PE (all *p* < 0.05). Conversely, no significant mediating effects were found for other parental feeding practices in the association between family socioeconomic factors and childhood overweight or obesity. However, socioeconomic factors of the family were positively correlated with scores on RST patterns (*β* = 0.53, *p* < 0.05). Additionally, CN (*β* = 0.04, *p* < 0.001) and PCW (*β* = 0.06, *p* < 0.001) were both positively associated with children’s overweight and obesity (Table 4).

## 4. Discussion

Contrary to our initial hypothesis, this longitudinal study found no significant associations between parental feeding practices and children’s body composition indices (FMI/FFMI) over a 2-year follow-up period. The secondary analyses revealed that certain parental feeding practices (e.g., perceived child weight and concern about child weight) were associated with an increased risk of central obesity in children. However, RST and FR were not associated with children’s body composition indicators [30,31]. In fact, our study did not replicate our hypothesis about the association between parental feeding practices and main outcomes (FMI/FFMI). This null finding is important and deserves further exploration.

We supposed that parental feeding practices were associated with children’s body composition indicators. Nevertheless, this longitudinal study found no significant associations between parental feeding practices and changes in children’s body composition (FMI/FFMI), suggesting that these practices may not exert a direct effect on fat mass or lean mass distribution in school-aged children. These null findings suggest that parental feeding behaviors may not be a strong or direct predictor of fat mass distribution in children, particularly after adjusting for confounders and using conservative statistical corrections (e.g., Bonferroni). This insight challenges the widespread assumption that modifying parental feeding styles alone can meaningfully alter children’s adiposity. The null associations between parental feeding practices and body composition (FMI/FFMI) may reflect methodological and biological factors. First, BIA-based measures have inherent limitations in detecting subtle fat mass changes, particularly in children [32]. Although BIA is practical for large-scale studies, it relies on weight-based equations and may lack precision in detecting small changes in fat mass distribution, particularly in children undergoing rapid growth [9]. Some more sensitive methods such as dual-energy X-ray absorptiometry (DXA) will be more accurate. Second, the 2-year follow-up may be insufficient to capture the long-term effects of feeding practices on fat partitioning, especially given the dominant influence of growth and puberty on fat-free mass development [33]. Additionally, feeding practices like pressure to eat may transiently affect caloric intake but not long-term fat partitioning, especially when confounded by unmeasured factors (e.g., physical activity). Prior studies have linked body composition to diet changes and physical activity [34,35,36]. Our findings could not confirm this because parental feeding practices may transiently influence children’s caloric intake but not necessarily alter long-term body composition. For example, Derks found that restrictive feeding did not predict changes in fat mass index in children, suggesting that genetic or metabolic factors may play a more dominant role in body composition development [37]. Finally, previous studies have found that parental feeding practices were associated with BMI or WHtR [5]; our null results for FMI/FFMI suggest that these practices may influence overall weight status rather than specific fat or lean mass distribution. This aligns with evidence that BMI is a crude measure of adiposity and may not fully capture metabolic risks associated with body composition. Moreover, it underscores the importance of acknowledging and reporting null results, as they help refine theoretical models and redirect future research toward potentially more proximal influences on child body composition, such as genetics, hormonal regulation, or physical activity.

Our findings are consistent with current literature, indicating that concern about child weight and perceived child weight were associated with central obesity in children, regardless of gender or parental education level [38]. Similar to our findings, a study in Chinese megacities reported that concern was positively associated with central obesity, suggesting that feeding practices may differentially impact central obesity compared to obesity [5]. Previous studies have shown that concern about child weight and dietary intake leads to undermining health-promoting parenting practices [39,40]. A potential bidirectional relationship has been suggested: parents may increase concern in response to children’s picky eating, while children subjected to such pressure may experience reduced enjoyment of eating and become more selective in their food choices [41]. Notably, while concern about child weight and perceived child weight were associated with central obesity, these associations may reflect parental reactions rather than causal influences. Parents of children with higher central obesity may develop heightened concern or monitoring behaviors in response to their child’s weight status, rather than these practices directly contributing to obesity. Longitudinal designs cannot disentangle this bidirectional relationship, and experimental studies are needed to test causal pathways. These associations should be interpreted with caution due to the possibility of reverse causality.

In this study, the mediation model examining the relationship between SES and childhood overweight/obesity indicated that this association was mediated exclusively by pressure to eat [42]. A positive correlation has been observed between SES and childhood overweight/obesity. SES indicators, including parental education level, occupation, and household income, may contribute to this correlation through the combined effects of parents’ health literacy, nutritional literacy, and family economic status, which influence feeding practices and subsequently, directly or indirectly, affect children’s dietary habits and lifestyles [43]. This suggests that family socioeconomic status, along with parents’ health literacy and feeding practices, plays a role in shaping children’s dietary patterns, managing weight status, and preventing childhood obesity. Parental feeding practices showed limited associations with children’s body composition changes. It is important to acknowledge that, given the observational nature of this study, the observed associations do not infer causality. The longitudinal design allows for tracking temporal relationships but cannot establish definitive causal pathways between parental feeding practices and children’s body composition and weight status. Feeding practices are influenced by her child’s weight status rather than causing subsequent weight gain. Future research should aim to validate potential bidirectional pathways and clarify underlying mechanisms.

This study has several limitations. First, the reliance on BIA for body composition measurement may lack precision compared to gold-standard methods (e.g., DXA), particularly in pediatric populations with rapid growth. Future studies should combine multi-compartment models with longer follow-ups to capture subtle changes. Second, the reliance on self-reported data measures of parental feeding practices may introduce biases, including social-desirability bias or recall bias. Future studies could incorporate objective measures to complement self-reported data. Third, the observed associations between parental feeding practices and children’s central obesity may involve reverse causality, and it remains unclear whether parental feeding practices are a cause or a consequence. Therefore, the causal pathway in this study is still uncertain.

This study also has several strengths. First, the sample size, derived from repeated measurements in a longitudinal survey, enables a more accurate capture of the impact of temporal changes on children’s body composition and their associated influencing factors. Second, given the limited number of studies focusing on the relationship between parental feeding practices and children’s body composition, this research provides important insights into the role of parents—as primary caregivers—in shaping children’s growth, development, and body composition. Finally, by focusing on children’s body composition, this study offers a more precise positioning and analysis of children’s nutritional health, as BMI alone may not directly estimate or quantify fat content, thereby reducing the accuracy of the research.

## 5. Conclusions

This longitudinal study found no significant associations between parental feeding practices and children’s body composition (FMI/FFMI), suggesting parental feeding practices may not directly influence fat or lean mass in school-aged children. Instead, observed links between parental feeding practices and children’s central obesity likely reflect reactive, rather than causal, responses. These findings challenge conventional assumptions and highlight the need for causally oriented research to clarify the directionality of parent-child influences and to identify more direct targets for obesity prevention.

## Figures and Tables

**Table 1:** Baseline characteristics of children and their parents in parental feeding practices in Luohu district, Shenzhen in 2021 (*n* = 620).

	MN ^a^		PE ^a^		RST ^a^		PCW ^a^		CN ^a^		PPW ^a^		FR ^a^	
	Low	High	Low	High	Low	High	Low	High	Low	High	Low	High	Low	High
Age (year)	8.52 ± 0.37	8.55 ± 0.38	8.52 ± 0.38	8.55 ± 0.36	8.51 ± 0.37	8.57 ± 0.37	8.53 ± 0.37	8.53 ± 0.39	8.54 ± 0.37	8.53 ± 0.37	8.54 ± 0.37	8.50 ± 0.38	8.54 ± 0.37	8.52 ± 0.37
BMI (kg/m^2^)	16.53 ± 3.00	16.96 ± 3.45	16.44 ± 3.53	15.82 ± 2.49 **	16.49 ± 2.94	17.10 ± 3.59 *	15.70 ± 2.25	19.63 ± 3.75 **	15.67 ± 2.34	18.30 ± 3.68 **	16.45 ± 3.05	18.16 ± 3.67 **	16.76 ± 3.20	16.69 ± 3.24
WHtR (cm)	0.44 ± 0.05	0.45 ± 0.06	0.46 ± 0.06	0.44 ± 0.05 **	0.44 ± 0.05	0.45 ± 0.06	0.43 ± 0.04	0.49 ± 0.07 **	0.43 ± 0.05	0.47 ± 0.06 **	0.44 ± 0.05	0.46 ± 0.06 *	0.45 ± 0.05	0.45 ± 0.06
Overweight/obesity (%) ^b^	23.58	29.82	35.24	15.13 **	24.60	29.34	12.88	64.81 **	13.75	45.38 **	23.51	41.58 **	26.92	25.89
Obesity (%) ^b^	13.13	17.19	21.49	6.64 **	11.90	19.83 *	4.59	44.44 **	5.39	29.32 **	12.52	27.72 **	14.79	15.25
Central obesity (%) ^c^	22.69	23.16	29.23	14.76 **	21.16	25.62	12.88	51.23 **	12.94	37.75 **	21.00	32.67 *	22.49	23.40
Maternal BMI	22.07 ± 3.55	21.63 ± 2.90	21.99 ± 3.16	21.70 ± 3.41	21.78 ± 3.07	22.00 ± 3.56	21.70 ± 3.22	22.34 ± 3.37 *	21.61 ± 3.12	22.24 ± 3.46 *	21.68 ± 3.16	22.82 ± 3.66 *	21.86 ± 3.03	21.87 ± 3.55
Paternal BMI	24.08 ± 3.20	24.05 ± 3.05	24.26 ± 3.01	23.82 ± 3.27	23.92 ± 3.08	24.29 ± 3.21	23.83 ± 3.16	24.73 ± 2.96 *	23.58 ± 2.99	24.79 ± 3.20 **	24.02 ± 3.10	24.31 ± 3.28	24.04 ± 2.83	24.09 ± 3.46

Abbreviations: MN: monitoring; PE: pressure to eat; RST: restriction; PCW: perceived child weight; CN: concern about child weight; PPW: perceived parent weight; FR: food as reward. Notes: ^a^: The scores on the seven dimensions of parental feeding practices were categorized into low and high groups according to median scores, and the characteristics across medians for specific parental feeding practices were tested using chi-square tests for categorical variables and analysis of variance (ANOVA) for continuous variables. ^b^: Overweight and obesity were defined using gender- and age-specific BMI cut-off points according to the Chinese national standard “WS/T586-2018 Screening for Overweight and Obesity in School-age Children and Adolescents.” ^c^: Central obesity was defined as a waist-to-height ratio (WHtR) ≥ 0.48. *: *p* < 0.05; **: *p* < 0.001.

**Table 2:** Descriptive analysis of outcome indicators at baseline and across two follow-up time points.

	T1 (n = 620)	T2 (n = 620)	T3 (n = 620)	Variability Within Individuals (T2 and T1)	Variability Within Individuals (T3 and T2)	Sex	
						Boys (n = 336)	Girls (n = 284)
Main outcome							
FMI (kg/m^2^)	3.11 ± 2.42	3.94 ± 3.09	3.99 ± 3.08	0.83 ± 1.18	0.05 ± 1.19	3.33 ± 0.15	2.85 ± 0.11 *
FFMI (kg/m^2^)	13.64 ± 1.03	13.84 ± 1.05	14.13 ± 1.13	0.21 ± 0.59	0.28 ± 0.62	14.01 ± 0.05	13.19 ± 0.06 **
Secondary outcome							
Overweight/obesity (%)	26.45	29.35	27.10	2.90	−2.25	32.44	19.37 **
Central obesity (%)	22.90	27.10	28.23	4.20	1.13	30.95	13.38 **

Abbreviations: FMI: fat mass index; FFMI: fat-free mass index. Notes: T1: Baseline, T2: first follow-up, T3: second follow-up. *, *p* < 0.05; **, *p* < 0.001.

**Table 3:** Longitudinal associations between parental feeding practices and children’s body composition and obesity-related indicators (3 rounds of repeated measures, 2021–2023).

	MN		PE		PCW		RST		CN		PPW		FR	
	*β*/OR (95%CI)	*p*	*β*/OR (95%CI)	*p*	*β*/OR (95%CI)	*p*	*β*/OR (95%CI)	*p*	*β*/OR (95%CI)	*p*	*β*/OR (95%CI)	*p*	*β*/OR (95%CI)	*p*
Main outcome														
FMI	0.04 (0.003, 0.07)	0.030	−0.01 (−0.04, 0.02)	0.602	0.01 (−0.05, 0.08)	0.635	−0.01 (−0.03, 0.02)	0.615	−0.003 (−0.05, 0.04)	0.897	0.01 (−0.01, 0.11)	0.114	−0.05 (−0.10, −0.002)	0.040
FFMI	0.01 (−0.003, 0.03)	0.123	−0.01 (−0.02, 0.01)	0.383	−0.01 (−0.04, 0.02)	0.513	−0.003 (−0.01, 0.01)	0.573	−0.002 (−0.02, 0.02)	0.881	0.03 (0.01, 0.06)	0.016	−0.01 (−0.03, 0.01)	0.300
Secondary outcome														
Overweight/obesity	1.12 (1.03, 1.23)	0.009	0.95 (0.88, 1.03)	0.218	1.26 (1.07, 1.49)	0.006	0.99 (0.94, 1.06)	0.530	1.18 (1.06, 1.31)	0.003	0.99 (0.85, 1.17)	0.605	0.94 (0.83, 1.07)	0.342
Central obesity	1.14 (1.06, 1.23)	<0.001	0.98 (0.91, 1.05)	0.525	1.33 (1.16, 1.51)	<0.001	1.02 (0.97, 1.07)	0.438	1.16 (1.07, 1.27)	<0.001	1.15 (1.01, 1.31)	0.017	0.98 (0.89, 1.09)	0.571

Abbreviations: MN: monitoring; PE: pressure to eat; RST: restriction; PCW: perceived child weight; CN: concern about child weight; PPW: perceived parent weight; FR: food as reward. FMI: fat mass index; FFMI: fat-free mass index. Note: Child sex, maternal and paternal BMI, and maternal and paternal education were adjusted as covariates in the mixed-effects model.

**Table 4:** Mediating effects of SES on childhood overweight and obesity.

	Direct Effect	*p*	Indirect Effect	*p*	Indirect Effect (%)	Path Coefficient		
						SES→Parental Feeding Practices ^a^	Parental Feeding Practices→Overweight/Obesity ^b^	SES→Overweight/Obesity ^c^
MN	0.04 (0.003, 0.01)	0.031	0.00 (−0.001, 0.01)	0.246	4.9	0.28	0.01	0.04 *
PE	0.04 (0.01, 0.08)	0.024	0.02 (0.01, 0.03)	0.002	26.5	−0.91 **	−0.02 **	0.06 *
RST	0.04 (0.01, 0.08)	0.025	0.00 (−0.004, 0.004)	0.969	0.2	0.53 *	0.00	0.04 *
CN	0.04 (0.01, 0.08)	0.023	−0.01 (−0.02, 0.001)	0.078	−33.8	−0.26	0.04 **	0.03
PCW	0.04 (0.004, 0.08)	0.032	−0.01 (−0.02, 0.01)	0.398	−13.6	−0.09	0.06 **	0.04
PPW	0.04 (0.01, 0.08)	0.019	0.00 (−0.003, 0.004)	0.804	1.1	−0.20 *	−0.002	0.04 *
FR	0.04 (0.01, 0.08)	0.025	0.00 (−0.004, 0.01)	0.855	1.1	0.37	0.00	0.04 *

Abbreviations: MN: monitoring; PE: pressure to eat; RST: restriction; PCW: perceived child weight; CN: concern about child weight; PPW: perceived parent weight; FR: food as reward. Note: * = *p* < 0.05, ** = *p* < 0.001. ^a^: a-path; ^b^: b-path; ^c^: c-path.

## Data Availability

The data can be retrieved from the corresponding author upon reasonable request due to (specify the reason for the restriction: e.g., privacy, legal or ethical reasons).

## Supplementary Materials

The following supporting information can be downloaded at https://www.mdpi.com/article/10.3390/nu17142255/s1, Table S1: Distribution of childhood overweight and obesity in relation to familial and social factors; Table S2: Distribution of childhood overweight and obesity among mediating factors.

## Abbreviations

The following abbreviations are used in this manuscript:

MN	Monitoring
PE	Pressure to eat
RST	Restriction
PCW	Perceived child weight
CN	Concern about child weight
PPW	Perceived parent weight
FR	Food as reward
BMI	Body mass index
WHtR	Waist-to-height ratio
FMI	Fat mass index
FFMI	Fat-free mass index
