# Supplementary material for: Analysis of the Longitudinal Association Between Parental Feeding Practices and Body Composition Among Children in Shenzhen
Source: Nutrients. 2025 Jul 8;17(14):2255. doi: 10.3390/nu17142255 (PMC12298824; doi:10.3390/nu17142255)
Supplement: Supplementary file 1 [file nutrients-17-02255-s001.zip › nutrients-3639247-supplementary.pdf]

Supplementary Table S1 Distribution of childhood overweight and obesity in relation to familial and social factors.

	Overall	Non-overweight/obesity （*n* =456）	Overweight/obesity （*n* =164）	*P* value
BMI of Mother	21.87±3.27	21.43±3.10	23.07±3.45	<0.001
BMI of Father	24.07±3.13	23.79±3.09	24.83±3.12	<0.001
Education of Mother				0.802
Junior High School or Below	93	76.34	23.66	
High School and Vocational Colleges	200	73.00	27.00	
University and Above	327	73.09	26.91	
Education of Father				0.591
Junior High School or Below	106	77.36	22.64	
High School and Vocational Colleges	200	72.00	28.00	
University and Above	314	73.25	26.75	
Occupation of Mother				0.007
Government Agencies, Enterprises, Professionals, and Clerical Staff	129	65.89	34.11	

Commercial Services and Life Service Personnel	201	70.15	29.85	
Military Personnel, Unclassified Occupations, and Agriculture-Related Occupations	290	79.11	20.69	
Occupation of Father				0.913
Government Agencies, Enterprises, Professionals, and Clerical Staff	148	72.30	27.70	
Commercial Services and Life Service Personnel	260	74.23	25.77	
Military Personnel, Unclassified Occupations, and Agriculture-Related Occupations	212	73.58	26.42	
Monthly Household Income (in 10,000 RMB)				0.230
<1	129	72.87	27.13	
1-2	241	77.18	22.82	
>2	250	70.40	29.60	

Supplementary Table S2 Distribution of childhood overweight and obesity among mediating factors.

	Overall	Non-overweight/obesity （n=456）	Overweight/obesity （n=164）	*P* value
Parental Feeding Practices				
MN (Continuous)	14.59±3.35	14.43±3.39	15.04±3.22	0.044
MN (Categorical)				0.079
Low	335	56.14	48.17	
High	285	43.86	51.83	
PE (Continuous)	12.79±3.54	13.23±3.45	11.55±3.50	<0.001
PE(Categorical)				<0.001
Low	329	49.56	75.00	
High	271	50.44	25.00	
RST (Continuous)	22.98±4.68	22.80±4.72	23.46±4.57	0.123
RST(Categorical)				0.192
Low	378	62.50	56.71	
High	242	37.50	43.29	
PCW (Continuous)	11.60±2.13	11.01±1.91	13.23±1.83	<0.001
PCW (Categorical)				<0.001
Low	458	87.50	35.98	
High	162	12.50	64.02	
CN (Continuous)	5.42±2.79	4.78±2.40	7.21±3.00	<0.001

CN (Categorical)				<0.001
Low	371	70.18	31.10	
High	249	29.82	68.90	
PPW (Continuous)	8.34±1.83	8.17±1.78	8.79±1.90	<0.001
PPW (Categorical)				<0.001
Low	519	87.06	74.39	
High	101	12.94	25.61	
FR (Continuous)	6.30±2.25	6.34±2.27	6.19±2.21	0.449
FR (Categorical)				0.771
Low	338	54.17	55.49	
High	282	45.83	44.51	

Abberivations: MN: monitoring; PE: pressure to eat; RST: restriction; PCW: perceived child weight; CN: concern about child weight; PPW: perceived parent weight; FR: food as reward.
